# Variation in Anticoagulant Recommendations by the Guidelines and Decision Tools among Patients with Atrial Fibrillation

**DOI:** 10.3390/healthcare3010130

**Published:** 2015-03-05

**Authors:** Anand Shewale, Jill Johnson, Chenghui Li, David Nelsen, Bradley Martin

**Affiliations:** 1 Division of Pharmaceutical Evaluation and Policy, University of Arkansas for Medical Sciences, Little Rock, AR 72205, USA; E-Mails: arshewale@uams.edu (A.S.); CLi@uams.edu (C.L.); 2 Department of Pharmacy Practice, University of Arkansas for Medical Sciences, Little Rock, AR 72205, USA; E-Mail: JohnsonJillT@uams.edu; 3 Department of Family Medicine, University of Arkansas for Medical Sciences, Little Rock, AR 72205, USA; E-Mail: NelsenDavidA@uams.edu

**Keywords:** atrial fibrillation, guidelines, decision tools, overuse, underuse, oral anticoagulants, warfarin, recommendations

## Abstract

Published atrial fibrillation (AF) guidelines and decision tools offer oral anticoagulant (OAC) recommendations; however, they consider stroke and bleeding risk differently. The aims of our study are: (i) to compare the variation in OAC recommendations by the 2012 American College of Chest Physicians guidelines, the 2012 European Society of Cardiology (ESC) guidelines, the 2014 American Heart Association (AHA) guidelines and two published decision tools by Casciano and LaHaye; (ii) to compare the concordance with actual OAC use in the overall study population and the population stratified by stroke/bleed risk. A cross-sectional study using the 2001–2013 Lifelink claims data was used to contrast the treatment recommendations by these decision aids. CHA_2_DS_2_-VASc and HAS-BLED algorithms were used to stratify 15,129 AF patients into nine stroke/bleed risk groups to study the variation in treatment recommendations and concordance with actual OAC use/non-use. The AHA guidelines which were set to recommend OAC when CHA_2_DS_2_-VASc = 1 recommended OAC most often (86.30%) and the LaHaye tool recommended OAC the least often (14.91%). OAC treatment recommendations varied considerably when stroke risk was moderate or high (CHA_2_DS_2_-VASc > 0). Actual OAC use/non-use was highly discordant (>40%) with all of the guidelines or decision tools reflecting substantial opportunities to improve AF OAC decisions.

## 1. Introduction

Atrial fibrillation (AF) is the most common type of cardiac arrhythmia which increases the risk of ischemic stroke 4 to 5 fold [1]. Oral anticoagulants (OACs) are more effective than aspirin in reducing the stroke risk, but are also associated with an increased bleeding risk. Therefore, anticoagulant recommendations depend upon balancing the expected benefit of stroke risk reduction against the increased harm from bleeding in patients with different factors that are prognostic for strokes and bleeding [2].

Several studies have shown that anticoagulant prescribing is often poorly related to patient stroke risk with underuse of anticoagulants in AF being more common, especially among elevated stroke risk patients [3,4,5]. One of the reasons for the under use of anticoagulants is the variation in physicians’ perceptions of the relative risk of stroke compared to bleeding by their specialty [6]. Physicians with a good personal experience with warfarin are more likely to prescribe warfarin; however, even these physicians prescribe warfarin in only half of their warfarin eligible AF patients [7]. Oral anticoagulants are not only underused but are also overused in the patients with low stroke risk [8]. 

Given the evidence of inappropriate OAC prescribing, various AF guidelines and decision support tools have been developed to aid clinicians considering oral anticoagulants for their AF patients. These decision aids include various clinical guidelines, model based decision tools and electronic medical record based apps and tools. However, these guidelines and decision support tools do not use the same stroke and bleed risk algorithms for treatment recommendations (Table S1) [9,10,11,12,13,14,15,16]. All the contemporary decision aids rely on the CHADS_2_ or the CHA_2_DS_2_-VASc scores to estimate stroke risk [17,18]. Some tools include formal bleed risk assessments including HAS-BLED and ATRIA, which are validated tools to estimate bleeding risk in AF patients [19,20]. The differences in the preferences for the stroke/bleed risk algorithm by these decision aids are due to the differences in the expert opinion and/or a lack of sufficient evidence supporting use of one approach over the other. The 2012 American College of Chest Physicians (CHEST) guideline recommendations are solely based on the stroke risk (CHADS_2_) scores as they have been extensively validated and easy for clinicians to remember, whereas the 2012 European Society of Cardiology (ESC) guidelines and the 2014 American College of Cardiology/American Heart Association Task Force on Practice Guidelines and the Heart Rhythm Society (AHA/ACC/HRS hereafter “AHA”) guideline recommendations are based on CHA_2_DS_2_-VASc [10,12,13]. Existing evidence suggest that the CHA_2_DS_2_-VASc has a better ability to identify patients who are at “truly low stroke risk” with a comparable overall discriminative ability in predicting ischemic stroke when compared to CHADS_2_ score [13]. Two published decision tools by Casciano *et al.* (hereafter “Casciano tool”) and LaHaye *et al.* (hereafter “Lahaye tool”) also include bleed risk scores (Casciano based on ATRIA, LaHaye based on HAS BLED) along with the stroke risk scores (Casciano based on CHADS_2_, LaHaye based on CHA_2_DS_2_-VASc) to derive their treatment recommendations. The tool developers did not provide a justification for choice of one bleed risk score over the other but this could be due to the lack of studies comparing the two bleed risk algorithms when the decision tools were developed [9,11].

Currently, there are no population-based studies that have compared the treatment recommendations by the AF guidelines and decision support tools. To address this, we empirically compared the treatment recommendations by the 2012 CHEST guidelines, the 2012 ESC guidelines, the 2014 AHA guidelines, the Casciano tool and the LaHaye tool [9,10,11,12,13]. To compare the acceptance of the guideline and decision tool recommendations in real world practice, we compared the concordance of actual OAC use/non-use between the decision aid recommendations. To understand the differences in the treatment recommendations by the decision aids and their concordance with actual OAC use/non-use at a more granular level, we also stratified our study population by their baseline stroke and bleed risk.

## 2. Methods

### 2.1. Study Design

A cross sectional study design using incident cases of AF from a 10% sample of the Lifelink claims database from 2001 to 2013 was used to contrast the treatment recommendations by these decision aids. The Lifelink claims data is nationally representative of the commercially insured population with respect to age, gender, geographic location and the type of insurance coverage. Separate files for inpatient claims, outpatient claims, prescription claims and eligibility data are available in the database. The inpatient and outpatient claim files include diagnosis codes in the International Classification of Disease, 9th Revision, Clinical Modification (ICD-9-CM) format as well as procedure codes in the Healthcare Common Procedure Coding System (HCPCS) format. The prescription claim files include data on national drug code (NDC) level and represents prescriptions covered by the insurance plan. The eligibility file was used to determine monthly enrolment/disenrollment in health plans. This study was approved by the University of Arkansas for Medical Sciences Institutional Review Board.

### 2.2. Study Subjects

The first primary diagnosis of AF was defined as the index diagnosis. Patients without any AF related claim within 12 months before the index diagnosis were defined as incident cases of AF. To rule out transient AF cases, we required patients to have at least two AF related claims separated by 30 days and have at least one outpatient claim with primary AF diagnosis in the post index period. Patients were also required to have continuous medical and pharmacy benefit eligibility for at least 12 months prior and three months after the index diagnosis. Table S2 shows a flowchart depicting the details of inclusion-exclusion criteria applied for the selection of final study sample.

### 2.3. Exposure Definition

The Lifelink claims database does not completely capture over-the-counter (OTC) aspirin use. Also, the newer oral anticoagulants were not available during most of the study time period. Therefore, the exposure definition was limited to oral anticoagulant (OAC) exposed and unexposed. A patient with at least one prescription claim for an oral anticoagulant (warfarin, dabigatran, apixaban or rivaroxaban) within 90 days of index diagnosis or at least one International Normalized Ratio/Prothrombin Time (INR/PT) claim within 90 days of the index diagnosis followed by a second INR/PT claim within 90 days of the first INR/PT claim date was considered as an OAC exposed patient. INR/PT test is often used as a proxy for warfarin exposure [21,22]. Patients who did not meet the definition of OAC exposure (including aspirin users) were considered as OAC unexposed patients.

### 2.4. Stroke Risk and Bleeding Risk

We calculated the stroke risk and bleeding risk using two stroke risk (CHADS_2_ and CHA_2_DS_2_-VASc) and two bleeding risk (ATRIA and HAS-BLED) algorithms. The operational definition to calculate stroke and bleeding risk scores were obtained from previous studies using administrative claims data [9,23,24,25].

### 2.5. Decision Aid Recommendations

The Casciano recommendations are based on the CHADS_2_ score, ATRIA score, patient characteristics (age and gender) to estimate the quality-adjusted life expectancy with warfarin/aspirin treatment [11]. The LaHaye tool offers treatment recommendations for aspirin, warfarin and newer oral anticoagulants based on the CHA_2_DS_2_-VASc score, HAS-BLED score, a treatment threshold (minimum stroke risk reduction after initiating antithrombotic therapy), a cost threshold (maximum cost that a patient is willing to pay per day in order to realize the specified treatment threshold) and bleed ratio (maximum number of major bleed events that a patient can endure to prevent one stroke event). Our analysis was restricted to warfarin and aspirin (ASA) recommendations of the LaHaye tool that were obtained by using fixed values of the treatment threshold (0.3%), the cost threshold ($0.50) and the bleed ratio (2:1) [9]. The CHEST guideline recommendations are based on the CHADS_2_ score while the ESC and the AHA guideline recommendations are based on the CHA_2_DS_2_-VASc algorithm [10,12,13]. The ESC guideline recommends caution for prescribing OAC when HAS BLED ≥ 3. To operationalize the ESC OAC recommendation, we set the ESC guideline to recommend withholding OAC in patients with CHA_2_DS_2_-VASc score = 1 and HAS BLED ≥ 3, and OAC in patients whose HAS-BLED score is less than 3 and CHA_2_DS_2_-VASc score = 1, and OAC in all patients when CHA_2_DS_2_-VASc score > 1 [13]. The AHA guideline does not offer a clear recommendation for OAC in patients when CHA_2_DS_2_-VASc score = 1. Therefore, we compared two scenarios: (i) conservative OAC AHA recommendations which assume that OAC is not recommended (AHA Conservative) and (ii) aggressive OAC AHA recommendations which assume that OAC is recommended (AHA Aggressive) when CHA_2_DS_2_-VASc score = 1 [10].

### 2.6. Main Independent Variable

#### Concordance Status

When a patient’s OAC exposure status was consistent with the decision aid’s recommendation (OAC recommended—OAC exposed, OAC not recommended—OAC unexposed), that patient was considered to be concordant with the treatment recommendation of the respective decision aid. We also looked at the underuse and overuse of OAC (Underuse: OAC recommended-OAC unexposed; Overuse: OAC not recommended-OAC exposed) separately.

### 2.7. Analyses

We compared the demographic characteristics of OAC exposed and unexposed. Based on the CHA_2_DS_2_-VASc and HAS-BLED scores, AF patients were classified into nine categories to study the variation in treatment recommendations by the decision aids and their concordance with actual OAC use/non-use. These nine categories reflected the nine possible combinations of low, moderate and high CHA_2_DS_2_-VASc (low—CHA_2_DS_2_-VASc = 0; moderate CHA_2_DS_2_-VASc = 1; high—CHA_2_DS_2_-VASc > 1) scores and HAS-BLED (low—HAS-BLED = 0; moderate HAS-BLED = 1 or 2; high—HAS-BLED ≥ 3) scores. The number of patients in each subgroup and their treatment recommendations were determined. In addition, the number and percent of patients discordant with the treatment recommendations by each decision aid were also determined. The decision aid recommendations and percent discordance with these recommendations were reported for the entire population to provide an overall acceptance of these decision aids. To assess the factors responsible for the difference in treatment recommendations and actual OAC use, we compared the characteristics of patients who were over-anticoagulated and under-anticoagulated based on the recommendations of each decision aid using a chi square test. Characteristics for comparison included age, gender, geographic region, prior stroke, hypertension, diabetes, heart failure, anemia, major bleed, renal failure, liver failure, vascular disease, alcohol use, medication use (anti-platelets, NASIDs, GI agents, antineoplastic agents, systemic corticosteroids), CHA_2_DS_2_-VASc score, CHADS_2_ score, ATRIA score and HAS-BLED score. All analyses were performed using SAS version 9.3 (SAS Institute Inc., Cary, NC, USA).

## 3. Results

**Figure 1:**
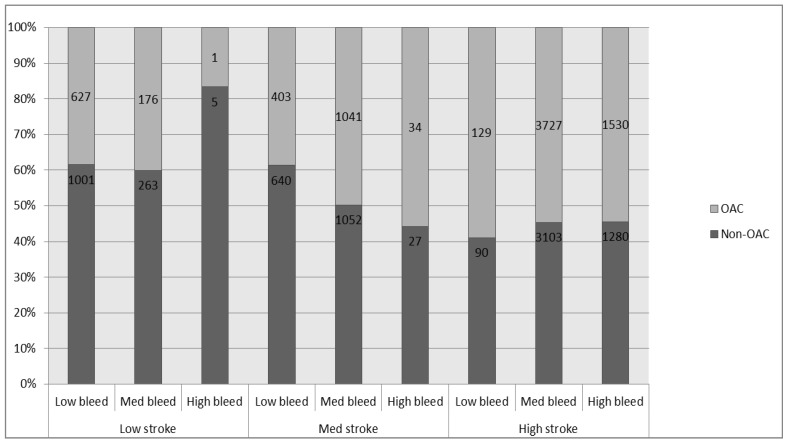
Actual OAC use: Number of patients that received and did not receive OAC stratified by stroke and bleeding risk scores. * Data shown are the number of patients who were exposed to OAC/non-OAC within 90 days after index diagnosis. Low stroke: CHA_2_DS_2_-VASc score = 0, Med stroke: CHA_2_DS_2_-VASc score = 1, high stroke: CHA_2_DS_2_-VASc score ≥ 2, Low bleed: HAS BLED score = 0, Med bleed: HAS BLED score = 1 or 2, high bleed: HAS BLED score ≥ 3).

A total of 15,129 incident AF patients met our inclusion-exclusion criteria (Table S2). Of these, 7668 (50.68%) were OAC exposed within first 90 days after the index diagnosis (Table 1). Compared to patients unexposed to OAC, a higher percentage of OAC exposed patients had CHA_2_DS_2_-VASc scores ≥2 (70.24% *vs.* 59.95%, *p* value < 001; Table 1 and Table S3). Also, the proportion of patients with moderate to high HAS-BLED score was higher among the OAC exposed group compared to the OAC unexposed group (84.89% *vs.* 76.79%, *p* value < 001). Actual OAC exposure was not always influenced by the baseline stroke and bleeding risks (Figure 1).

The AHA Aggressive guideline recommended OAC most often (86.30%) and the LaHaye tool recommended OAC the least often (14.91%; Table 2). None of the decision aids recommended OAC for patients when CHA_2_DS_2_-VASc = 0. However, OAC treatment recommendations varied considerably when the stroke risk was moderate or high (CHA_2_DS_2_-VASc > 0). For example, the LaHaye tool never recommended OAC for patients with CHA_2_DS_2_-VASc = 1 irrespective of bleeding risk, however, the ESC guidelines as we defined them universally recommended OAC for patients with CHA_2_DS_2_-VASc = 1 and a HAS-BLED < 3. For those with the highest stroke risk, the ESC and AHA guidelines recommended OAC in all patients with CHA_2_DS_2_-VASc score ≥ 2 irrespective of the HAS-BLED score, whereas the CHEST guidelines, Casciano and LaHaye tools recommended OAC between 22.88% and 95.57% of high stroke risk patients.

**Table 1:** Demographics characteristics of the study population.

Characteristics	OAC Exposed	OAC Unexposed	Total	*p* Value
**Number of patients (n %)**	7668 (50.68%)	7461 (49.32%)	15129	
**Mean age, y (SD)**	65.04 (11.82)	62.54 (14.84)	63.81 (13.45)	<0.001
	**N (%)**	**N (%)**	**N (%)**	
**Age Category, y**				<0.001
18–30	29 (0.38%)	146 (1.96%)	175 (1.16%)	
31–49	584 (7.62%)	1156 (15.49%)	1740 (11.50%)	
50–64	3453 (45.03%)	3092 (41.44%)	6545 (43.26%)	
65–74	1745 (22.76%)	1240 (16.62%)	2985 (19.73%)	
75–84	1570 (20.47%)	1429 (19.15%)	2999 (19.82%)	
≥85	287 (3.74%)	398 (5.33%)	685 (4.53%)	
**Gender**				<0.001
Male	4961 (53.16%)	4372 (46.84%)	9333 (61.69%)	
Female	2707 (35.30%)	3089 (41.40%)	5796 (38.31%)	
**Geographic Region**				<0.001
East	1591 (20.75%)	1474 (19.76%)	3065 (20.26%)	
Midwest	2656 (34.64%)	2454 (32.89%)	5110 (33.78%)	
South	2301 (30.01%)	2271 (30.44%)	4572 (30.22%)	
West	1120 (14.61%)	1262 (16.91%)	2382 (15.74%)	
**Comorbidities**				
Prior stroke or TIA	551 (7.19%)	476 (6.38%)	1027 (6.79%)	0.048
Hypertension	5177 (67.51%)	4263 (57.14%)	9440 (62.4%)	<0.001
Diabetes	1857 (24.22%)	1265 (16.95%)	3122 (20.64%)	<0.001
Heart failure	1506 (19.64%)	884 (11.85%)	2390 (15.8%)	<0.001
Anemia	731(9.53%)	864 (11.58%)	1595 (10.54%)	<0.001
History of any bleed	675 (8.80%)	732 (9.81%)	1407 (9.30%)	0.032
Renal impairment	493 (6.43%)	441 (5.91%)	934 (6.17%)	0.185
Liver failure	255 (3.33%)	258 (3.46%)	513 (3.39%)	0.652
Vascular disease	2377 (31.00%)	2202 (29.51%)	4579 (30.27%)	0.046
Antiplatelet agents	488 (6.36%)	403 (5.40%)	891 (5.89%)	0.011
NASIDs	1443 (18.82%)	1208 (16.19%)	2651 (17.52%)	<0.001
GI agents	1532 (19.98%)	1398 (18.74%)	2930 (19.37%)	0.053
Antineoplastic agents	178 (2.32%)	135 (1.81%)	313 (2.07%)	0.027
Systemic corticosteroids	1136 (14.81%)	939 (12.59%)	2075 (13.72%)	<0.001
Alcohol use	124 (1.62%)	119 (1.59%)	243 (1.61%)	0.913
**Stroke risk scores**				
***CHADS_2_ score***				<0.001
Low (score = 0)	1524 (19.87%)	2426 (32.52%)	3950 (26.11%)	
Medium (score = 1)	2676 (34.90%)	2510 (33.64%)	5186 (34.28%)	
High (score ≥ 2)	3468 (45.23%)	2525 (33.84%)	5993 (39.61%)	
***CHA_2_DS_2_-VASc score***				<0.001
Low (score = 0)	804 (10.49%)	1269 (17.01%)	2073 (13.70%)	
Medium (score = 1)	1478 (19.27%)	1719 (23.04%)	3197 (21.13%)	
High (score ≥ 2)	5386 (70.24%)	4473 (59.95%)	9859 (65.17%)	
**Bleeding risk scores**				
***ATRIA score***				0.002
Low (score ≤ 3)	6590 (85.94%)	6391 (85.66%)	12981 (85.80%)	
Medium (score = 4)	596 (6.29%)	401 (5.37%)	883 (5.84%)	
High (score ≥ 5)	596 (7.77%)	669 (8.97%)	1265 (8.36%)	
***HAS BLED score***				<0.001
Low (score = 0)	1159 (15.11%)	1731 (23.20%)	2890 (19.10%)	
Medium (score = 1 or 2)	4944 (64.48%)	4418 (59.21%)	9362 (61.88%)	
High (score ≥ 3)	1564 (20.41%)	1312 (17.58%)	2877 (19.02%)	
**Anticoagulant exposure after index date**				
Warfarin	5886 (76.76%)	-	5886 (38.91%)	
Dabigatran	756 (9.86%)	-	756 (5.00%)	
Rivaroxaban	178 (2.32%)	-	178 (1.18%)	
Apixaban	5 (0.07%)	-	5 (0.03%)	
Antiplatelet agents	365 (4.76%)	434 (5.82%)	799 (5.28%)	

SD, standard deviation, TIA: transient ischemic attack, NASIDs: Non-steroidal anti-inflammatory drugs, GI agents: Antacids, proton pump inhibitors, H2 receptor antagonist and other GI protectants, OAC/INR: use of oral anticoagulant or INR test with 90 days after index diagnosis.

The overall OAC exposure/non-exposure was most consistent with the Casciano tool (56.75%) and was least consistent with the LaHaye tool (49.94%; Table S4). The overall percent discordance with the decision aid recommendations was fairly consistent over the study period (Table S5). When the underuse of OAC was assessed using the nine stroke/bleed risk groups, underuse of OAC was similar between moderate and high stroke risk patients with OAC not being prescribed between 43.98% and 53.95% of patients recommended OAC (Table 3).

Most of the OAC unexposed patients who were recommended OAC by the decision aids had a high stroke risk profile (72.55%–100%; Table S6). Since none of the decision aids recommended OAC use among patients with a low stroke risk, the percent of patients who were over-anticoagulated (38.78%) was consistent across all the decision aids at CHA_2_DS_2_-VASc score = 0 (Table 4).

Among the OAC exposed patients (*n* = 7668), the decision aids recommended withholding the OAC therapy in 10.49%–84.68% of the patients depending on the decision aid. Most of the patients (35.14%–100%) who were exposed to OAC when not recommended had low to moderate stroke risk profiles (Table S7).

**Table 2:** Oral anticoagulation (OAC) recommendations by the decision aid.

Stroke Risk	Bleeding risk	AHA Aggressive	AHA Conservative	Casciano	CHEST	ESC	LaHaye
**Low (CHA_2_DS_2_-VASc score = 0)**	**Low (HAS-BLED score = 0)**	0	0	0	0	0	0
	n (%) = 1628 (10.76%)	(0.00%)	(0.00%)	(0.00%)	(0.00%)	(0.00%)	(0.00%)
	**Medium (HAS-BLED score = 1 or 2)**	0	0	0	0	0	0
	n (%) = 439(2.90%)	(0.00%)	(0.00%)	(0.00%)	(0.00%)	(0.00%)	(0.00%)
	**High (HAS-BLED score ≥ 3) **	0	0	0	0	0	0
	n (%) = 6 (0.04%)	(0.00%)	(0.00%)	(0.00%)	(0.00%)	(0.00%)	(0.00%)
	**Sub-total**	0	0	0	0	0	0
		(0.00%)	(0.00%)	(0.00%)	(0.00%)	(0.00%)	(0.00%)
**Medium (CHA_2_DS_2_-VASc score = 1)**	**Low (HAS-BLED score = 0) **	1043	0	193	196	1043	0
	n (%) = 1043(6.89%)	(100%)	(0.00%)	(18.50%)	(18.79%)	(100%)	(0.00%)
	**Medium (HAS-BLED score = 1 or2)**	2093	0	1371	1505	2093	0
	n (%) = 2093(13.83%)	(100%)	(0.00%)	(65.50%)	(71.91%)	(100%)	(0.00%)
	**High (HAS-BLED score ≥ 3)**	61	0	19	57	0	0
	n (%) = 61 (0.40%)	(100%)	(0.00%)	(31.15%)	(93.44%)	(0.00%)	(0.00%)
	**Sub-total**	3197	0	1583	1758	3136	0
		(100%)	(0.00%)	(49.51%)	(54.98%)	(98.09%)	(0.00%)
**High (CHA_2_DS_2_-VASc score ≥ 2)**	**Low (HAS-BLED score = 0)**	219	219	151	158	219	219
	n (%) = 219 (1.45%)	(100.00%)	(100.00%)	(68.95%)	(72.15%)	(100.00%)	(100.00%)
	**Medium (HAS-BLED score = 1 or 2)**	6830	6830	5819	6468	6830	1102
	n (%) = 6830 (45.15%)	(100.00%)	(100.00%)	(85.20%)	(94.70%)	(100.00%)	(16.13%)
	**High (HAS-BLED score ≥ 3)**	2810	2810	2210	2795	2810	935
	n (%) = 2810 (18.57%)	(100.00%)	(100.00%)	(78.65%)	(99.47%)	(100.00%)	(33.27%)
	**Sub-total**	9859	9859	8180	9421	9859	2256
		(100.00%)	(100.00%)	(82.96%)	(95.57%)	(100.00%)	(22.88%)
**Overall**	**15129 (100%)**	13056	9859	9763	11179	12995	2256
		(86.30%)	(65.17%)	(64.53%)	(73.89%)	(85.89%)	(14.91%)

Data is presented as n (%) for oral anticoagulant recommendations by each decision aid. AHA Aggressive: AHA guidelines (assuming that guideline recommends OAC at CHA_2_DS_2_-VASc = 1), AHA Conservative: AHA guidelines (assuming that guideline recommends non-OAC at CHA_2_DS_2_-VASc = 1), Casciano: Casciano tool, CHEST: CHEST guidelines, ESC: ESC guidelines, LaHaye: LaHaye tool.

**Table 3:** Underuse of OAC (Number of patients discordant with the OAC treatment recommendation by decision aids).

Stroke Risk	Bleeding Risk	AHA Aggressive	AHA Conservative	Casciano	CHEST	ESC	LaHaye
Low (CHA_2_DS_2_-VASc score = 0)*	**Sub-total**	ARNO	ARNO	ARNO	ARNO	ARNO	ARNO
**Medium (CHA_2_DS_2_-VASc score = 1)**	**Low (HAS-BLED score = 0)**	640 (61.36%)	ARNO	52 (26.94%)	54 (27.55%)	640 (61.36%)	ARNO
	**Medium (HAS-BLED score = 1 or 2)**	1052 (50.26%)	ARNO	661 (48.21%)	728 (48.37%)	1052 (50.26%)	ARNO
	**High (HAS-BLED score ≥ 3)**	27 (44.26%)	ARNO	8 (42.11%)	27 (47.37%)	ARNO	ARNO
	**Sub-total**	1719 (53.77%)	ARNO	721 (45.55%)	809 (46.02%)	1692 (53.95%)	ARNO
**High (CHA_2_DS_2_-VAS score ≥ 2) **	**Low (HAS-BLED score = 0)**	90 (41.10%)	90 (41.10%)	47 (31.13%)	48 (30.38%)	90 (41.10%)	90 (41.10%)
	**Medium (HAS-BLED score = 1 or 2)**	3103 (45.43%)	3103 (45.43%)	2578 (44.30%)	2904 (44.90%)	3103 (45.43%)	542 (49.18%)
	**High (HAS-BLED score ≥ 3)**	1280 (45.55%)	1280 (45.55%)	973 (44.03%)	1274 (45.58%)	1280 (45.55%)	449 (48.02%)
	**Sub-total**	4473 (45.37%)	4473 (45.37%)	3598 (43.98%)	4226 (44.86%)	4473 (45.37%)	1081 (47.92%)
**Overall**	**Total**	**6192 (47.42%)**	**4473 (48.01%)**	**4319 (44.23%)**	**5035 (45.04%)**	**6165 (47.44%)**	**1081 (47.92%)**

Data is presented as n (%) of patients who are not consistent with the OAC treatment recommendation by each decision aid, ARNO: Always Recommends Non-OAC. * Data not shown by bleed risk because no decision aids recommend OAC when CHA_2_DS_2_-VASc = 1 irrespective of HAS-BLED score. AHA Aggressive: AHA guidelines (assuming that guideline recommends OAC at CHA_2_DS_2_-VASc = 1), AHA Conservative: AHA guidelines (assuming that guideline recommends non-OAC at CHA_2_DS_2_-VASc = 1), Casciano: Casciano tool, CHEST: CHEST guidelines, ESC: ESC guidelines, LaHaye: LaHaye tool.

**Table 4:** Overuse of OAC (Number of patients discordant with the non-OAC treatment recommendation by decision aids).

Stroke Risk	Bleeding Risk	AHA Aggressive	AHA Conservative	Casciano	CHEST	ESC	LaHaye
**Low (CHA_2_DS_2_-VASc score = 0)**	**Low (HAS-BLED score = 0)**	627 (38.51%)	627 (38.51%)	627 (38.51%)	627 (38.51%)	627 (38.51%)	627 (38.51%)
	**Medium (HAS-BLED score = 1 or 2)**	176 (40.09%)	176 (40.09%)	176 (40.09%)	176 (40.09%)	176 (40.09%)	176 (40.09%)
	**High (HAS-BLED score ≥ 3)**	1 (16.67%)	1 (16.67%)	1 (16.67%)	1 (16.67%)	1 (16.67%)	1 (16.67%)
	**Sub-total**	804 (38.78%)	804 (38.78%)	804 (38.78%)	804 (38.78%)	804 (38.78%)	804 (38.78%)
**Medium (CHA_2_DS_2_-VASc score = 1**	**Low (HAS-BLED score = 0)**	ARO	403 (38.64%)	262 (30.82%)	261 (30.81%)	ARO	403 (38.64%)
	**Medium (HAS-BLED score = 1 or 2)**	ARO	1041 (49.74%)	331 (45.84%)	264 (44.90%)	ARO	1041 (49.74%)
	**High (HAS-BLED score ≥ 3)**	ARO	34 (55.74%)	23 (54.76%)	4 (100.00%)	34 (55.74%)	34 (55.74%)
	**Sub-total**	ARO	1478 (46.23%)	616 (38.17%)	529 (36.76%)	34 (55.73%)	1478 (46.23%)
**High (CHA_2_DS_2_-VASc score ≥ 2) **	**Low (HAS-BLED score = 0)**	ARO	ARO	25 (36.76%)	19 (31.15%)	ARO	ARO
	**Medium (HAS-BLED score = 1 or 2)**	ARO	ARO	486 (48.07%)	163 (45.03%)	ARO	3167 (55.29%)
	**High (HAS-BLED score ≥ 3)**	ARO	ARO	293 (48.83%)	9 (60.00%)	ARO	1044 (55.68%)
	**Sub-total**	ARO	ARO	804 (47.88%)	191 (43.61%)	ARO	4211 (55.38%)
**Overall**	**Total**	**804 (38.78%)**	**2282 (43.30%)**	**2224 (41.45%)**	**1564 (39.59%)**	**838 (39.27%)**	**6493 (50.43%)**

Data is presented as n (%) of patients who are not consistent with the non- OAC treatment recommendation by each decision aid. ARO: Always recommends OAC. AHA Aggressive: AHA guidelines (assuming that guideline recommends OAC at CHA_2_DS_2_-VASc = 1), AHA Conservative: AHA guidelines (assuming that guideline recommends non-OAC at CHA_2_DS_2_-VASc = 1), Casciano: Casciano tool, CHEST: CHEST guidelines, ESC: ESC guidelines, LaHaye: LaHaye tool.

## 4. Discussion

There is a considerable variation in the treatment recommendations by the decision aids. The AHA and CHEST guidelines base their recommendations exclusively on the stroke risk algorithms, and these recommend OAC to more patients (65.17%–86.30%) compared to the LaHaye (14.91%) and Casciano (64.53%) tools which formally incorporate bleeding risk. The decision tools/guidelines which are based on the same stroke risk algorithm have more similarity in the treatment recommendations compared to those which are based on different stroke risk algorithms. For example, the treatment recommendations by the CHEST guidelines are more similar to the Casciano tool recommendations, both of which are based on the CHADS_2_ algorithm compared to the CHA_2_DS_2_VASc based ESC guideline and LaHaye tool. However, the treatment recommendations by the decisions aids based on the same stroke risk algorithm also differ depending on the weight given to the bleeding risk by the respective decision aids. For example, the LaHaye tool incorporates HAS-BLED score (0 to 7) at all levels of CHA_2_DS_2_VASc score (0 to 9) which is different than the ESC guidelines in which we only considered bleed risk to be a factor when CHA_2_DS_2_VASc = 1, which led to a 70.98% difference in OAC recommendations.

The treatment recommendations by the decision aids varied considerably among patients with moderate to high stroke risk. The stroke risk algorithm used and the weight given to the bleeding risk by respective decision aids mostly influences this variation. As the CHA_2_DS_2_-VASc algorithm accounts for additional stroke factors (vascular disease, female gender and age between 65 and 74 years) [18], the decision aids based on CHA_2_DS_2_-VASc classifies a higher proportion of patients in moderate and particularly in high stroke risk groups compared to the decision aids which are based on the CHADS_2_ algorithm. Although the LaHaye and the Casciano decision support tools consider bleeding risk algorithms in rendering OAC recommendations, both tools are based on different stroke risk and bleeding risk algorithms. Furthermore, the LaHaye tool recommendations are also influenced by bleed, treatment and cost thresholds while the Casciano tool recommendations are based on quality-adjusted life survival which incorporates demographic factors in addition to stroke and bleeding risk to render warfarin/aspirin treatment recommendations [9,11].

Despite the recommendation of withholding OAC in patients with CHA_2_DS_2_-VASc = 0 by all the decision aids, we found considerable utilization of OAC in these patients. Nearly 40% of CHA_2_DS_2_-VASc = 0 patients were OAC exposed which is similar to the 43.1% found by Lip *et al.* suggesting that overutilization is a widespread and persistent problem [26].

Our study confirms that OACs are underused in patients with elevated stroke risk [3,4,5]. We found that depending on the decision aid, between 45% and 48% were NOT anticoagulated when recommended. Although the use of OACs reduces the risk of stroke in all patients, it also increases the risk of hemorrhagic events [11]. Therefore, both overuse and underuse can be viewed as a concern in terms of AF related adverse events. When compared with the “real world” data, we found that the direction of OAC misuse was in both directions, patients who were OAC treatment eligible (moderate-high stroke risk) were often not anticoagulated while patients who were not eligible for OAC treatment (moderate–high bleeding risk with low stroke risk) were anticoagulated. The reason for this discrepancy between the actual OAC use and guideline recommendations could be attributed to the difference in physicians’ perceptions and empirical algorithms for risk assessment of stroke/bleed events. Hypertension, heart failure, age and diabetes have a smaller influence on physician assigned stroke risk while prior stroke, severe AF symptoms and not living independently have a higher influence on physician assigned stroke risk when compared to empirical stroke risk algorithms. [27] Our data had similar findings; patients who were under-anticoagulated had a high proportion of hypertensive patients (68.71%–84.67%), females (43.88%–72.43%), patients with vascular disease (35.56%–75.39%) and diabetes (20.43%–44.96%).

## 5. Limitations

The newer oral anticoagulants were not available over most of the study time thereby limiting the study to more general OAC/non-OAC comparisons. We relied on the prescription claims supplemented by INR test to identify OAC exposure and 6.29% of patients were considered OAC exposed by INR tests alone and a portion of these patients may be misclassified. Furthermore, this study could not assess the degree of guideline concordance with aspirin recommendations. The database used for this study does not contain information on lab values for the INR/PT test, which is one of the risk factors to calculate the HAS-BLED score. The lack of information about this risk factor might underestimate the bleeding risk determined by HAS-BLED for those that are anticoagulated with warfarin over time. However, the aim of our study was to assess the appropriateness of initial anticoagulation, and the lack of information on labile INR would not significantly influence our study findings. When administrative claims data are used, coding errors could influence study measures. The accuracy of identifying the comorbid conditions used to calculate stroke/bleed risk scores using the administrative database has been previous validated (crude agreement: 78%–96%) [23,28] however not all study measures have known validity [11,24,29]. Finally, the data is representative of a commercially insured population and the study findings may not be applicable for other populations.

## 6. Conclusions

Real world OAC prescribing is frequently discordant with all the common contemporary guidelines and decision aids with both overuse and underuse of OAC being detected. There is a considerable variability in the OAC treatment recommendations by the AF guidelines and decision tools when the ischemic stroke risk is moderate or high. Given the variability in guideline recommendations, empirical evidence is needed to compare the outcomes of OAC treatment recommendations for these decision aids.

## Supplementary Files

Supplementary File 1
