# Supplementary material for: Variation in Anticoagulant Recommendations by the Guidelines and Decision Tools among Patients with Atrial Fibrillation
Source: Healthcare (Basel). 2015 Mar 5;3(1):130–45. doi: 10.3390/healthcare3010130 (PMC4934528; doi:10.3390/healthcare3010130)
Supplement: Supplementary File 1 [file healthcare-03-00130-s001.pdf]

Supplemental Materials

Table S1. Treatment recommendations by the decision aids.

Decision aid	Stroke risk algorithm used	Bleed risk algorithm used	Additional factors	Recommendations
AHA guidelines	CHA_2DS_2-VASc			Non-OAC if CHA2DS2-VASc = 0; Non-OAC or OAC if CHA2DS2-VASc = 1; OAC if CHA2DS2-VASc ≥ 2
Casciano tool	$CHADS_2$	ATRIA	Age and gender	Case specific
CHEST guidelines	$CHADS_2$			Non-OAC, if CHADS2 = 0; OAC if CHADS2 ≥ 1
ESC guidelines	CHA_2DS_2-VASc	Warrants a caution if HAS-BLED score ≥ 3		Non-OAC if CHA2DS2-VASc = 0; Non-OAC or OAC if CHA2DS2-VASc = 1; OAC if CHA2DS2-VASc ≥ 2
LaHaye tool	CHA_2DS_2-VASc	HAS-BLED	Treatment threshold, cost threshold and bleed ratio	Case specific

AHA guideline: the 2014 American College of Cardiology/American Heart Association Task Force on Practice Guidelines, Casciano tool: Decision tool developed by Casciano *et al.*, CHEST guidelines: the 2012 American College of Chest Physicians (CHEST) guidelines, ESC guidelines: the 2012 European Society of Cardiology (ESC) guidelines, LaHaye tool: Decision tool developed by LaHaye *et al.*

Table S2. Inclusion and exclusion criteria.

**AF Population of Lifelink data**<br>Population (January 1st, 2001 through June 30th, 2013)	# of Enrollees
**Inclusion criteria:**	
Primary/secondary AF diagnosis (ICD-9-CM code 427.31) between January 1st, 2002 through March 31th, 2013 (inpatient and outpatient files)	222520
Primary AF diagnosis (ICD-9-CM code 427.31) between January 1st, 2002 through March 31th, 2013 (inpatient, outpatient or ancillary files)	135618
At least 12 months of continuous enrollment in the pre-index period	83643
At least two AF diagnoses in the in the inpatient or outpatient files	65045
At least one diagnosis of AF at least 30 days after initial diagnosis in the study period	55320
At least one primary diagnosis of AF in the outpatient setting in the study period	55213
At least 18 years of age at the time of initial primary diagnosis of Atrial Fibrillation in the study period	54805
At least 12 months of pharmacy benefits in the pre-index period	39251
At least 3 months of pharmacy benefits in the post-index period	38434
**No. of patients after applying the exclusion criteria:**	
Patients with perioperative/ transient atrial fibrillation	38434
Patients with inpatient or outpatient diagnosis of mitral stenosis or prosthetic heart valve	36571
Patients with concomitant hyperthyroidism or thyrotoxicosis	35842
Patients with a diagnosis of AF in the pre-index period	19719
Patients with a prescription claims for oral anticoagulants	17031
Patients with a claim for INR/PT test	15603
Patients who have an thromboembolic event, intracranial hemorrhagic or extra-cranial hemorrhagic event within 90 days of their index AF diagnosis	15413
Potential duplicates across two datasets (if the index date, year of birth, gender, patient region and first date of enrolment are same)	15129
**Final sample size**	15129

Table S3. Stroke risk and bleeding risk scores.

Score	CHADS2 N (%)	CHA2DS2-VASc N (%)	ATRIA N (%)	HAS BLED N (%)
0	3950 (26.11%)	2073 (13.70%)	4281 (28.30%)	2890 (19.10%)
1	5186 (34.28%)	3197 (21.13%)	5465 (36.12%)	4754 (31.42%)
2	3620 (23.93%)	3022 (19.97%)	1202 (7.95%)	4608 (30.46%)
3	1530 (10.11%)	2578 (17.04%)	2033 (13.44%)	2053 (13.57%)
4	591(3.91%)	2035 (13.45%)	883 (5.84%)	652 (4.31%)
5	202 (1.34%)	1224 (8.09%)	237 (1.57%)	149 (0.98%)
6	50 (0.33%)	628 (4.15%)	545 (3.60%)	21 (0.14%)
7		260 (1.72%)	253 (1.67%)	2 (0.01%)
8		97 (0.64%)	37 (0.24%)	
9		15 (0.1%)	150 (0.99%)	
10			43 (0.28%)	

Table S4. Number of patients concordant with the OAC or non-OAC treatment recommendation by decision aids.

Stroke risk	Bleeding risk	AHA Aggressive	AHA Conservative	Casciano	CHEST	ESC	LaHaye
Low (CHA_2DS_2-VASc score = 0)	Low (HAS-BLED score = 0)	1001 (61.49%)	1001 (61.49%)	1001 (61.49%)	1001 (61.49%)	1001 (61.49%)	1001 (61.49%)
	Medium (HAS-BLED score = 1 or 2)	263 (59.91 %)	263 (59.91%)	263 (59.91 %)	263 (59.91%)	263 (59.91 %)	263 (59.91 %)
	High (HAS-BLED score ≥ 3)	5 (83.33%)	5 (83.33%)	5 (83.33%)	5 (83.33%)	5 (83.33%)	5 (83.33%)
	Sub-total	1269 (61.21%)	1269 (61.21%)	1269 (61.21%)	1269 (61.21%)	1269 (61.21%)	1269 (61.21%)
Medium (CHA_2DS_2-VASc score = 1)	Low (HAS-BLED score = 0)	403 (38.64%)	640 (61.36%)	729 (69.89%)	728 (69.80%)	403 (38.64%)	640 (61.36%)
	Medium (HAS-BLED score = 1 or 2)	1041 (49.74%)	1052 (50.26%)	1101 (52.60%)	1101 (52.60%)	1041 (49.74%)	1052 (50.26%)
	High (HAS-BLED score ≥ 3)	34 (55.74%)	27 (44.26%)	30 (49.18%)	30 (49.18%)	27 (44.26%)	27 (44.26%)
	Sub-total	1478 (46.23%)	1719 (53.77%)	1860 (58.18%)	1859 (58.15%)	1471 (46.01%)	1719 (53.78%)
High (CHA_2DS_2-VASc score ≥ 2)	Low (HAS-BLED score = 0)	129 (58.90%)	129 (58.90%)	147 (67.12%)	152 (69.41%)	129 (58.90%)	129 (58.90%)
	Medium (HAS-BLED score = 1 or 2)	3727 (54.57%)	3727 (54.57%)	3766 (55.14%)	3763 (55.10%)	3727 (54.57%)	3121 (45.70%)
	High (HAS-BLED score ≥ 3)	1530 (54.45%)	1530 (54.45%)	1544 (54.95%)	1527 (54.34%)	1530 (54.45%)	1317 (46.87%)
	Sub-total	5386 (54.63%)	5386 (54.63%)	5457 (55.35%)	5442 (55.20%)	5386 (54.63%)	4567 (46.32%)
	Total	8133 (53.76%)	8374 (55.35%)	8586 (56.75%)	8570 (56.65%)	8126 (53.71%)	7555 (49.94%)

Data is presented as n (%) of patients who are consistent with the treatment recommendation (OAC/non-OAC) by each decision aid. AHA Aggressive: AHA guidelines (assuming that guideline recommends OAC at CHA_2DS_2-VASc = 1), AHA Conservative: AHA guidelines (assuming that guideline recommends non-OAC at CHA_2DS_2-VASc = 1), Casciano: Casciano tool, CHEST: CHEST guidelines, ESC: ESC guidelines, LaHaye: LaHaye tool.

Table S5. Percent discordant with OAC/non-OAC recommendations of the decision aids stratified by index year.

Index year	AHA Aggressive, n (%)	AHA Conservative, n (%)	Casciano, n (%)	CHEST, n (%)	ESC, n (%)	LaHaye, n (%)
**2002**	73 (42.44)	79 (45.93)	62 (36.05)	66 (38.37)	74 (43.02)	89 (51.74)
**2003**	47 (40.52)	49 (42.24)	49 (42.24)	46 (39.44)	47 (40.52)	70 (60.34)
**2004**	218 (44.95)	215 (44.33)	205 (42.27)	203 (41.86)	219 (45.15)	250 (51.55)
**2005**	216 (42.02)	222 (43.19)	210 (40.86)	211 (41.05)	217 (42.22)	287 (55.84)
**2006**	255 (46.45)	283 (51.55)	268 (48.82)	265 (48.27)	256 (46.63)	287 (52.28)
**2007**	757 (47.14)	754 (46.95)	713 (44.40)	723 (45.02)	760 (47.32)	813 (50.62)
**2008**	1207 (46.39)	1147 (44.08)	1137 (43.70)	1123 (43.16)	1210 (46.50)	1294 (49.73)
**2009**	1348 (47.33)	1305 (45.82)	1225 (43.01)	1247 (43.79)	1345 (47.23)	1435 (50.39)
**2010**	1170 (46.06)	1113 (43.82)	1074 (42.28)	1094 (43.07)	1165 (45.87)	1240 (48.82)
**2011**	1012 (47.76)	943 (44.50)	938 (44.27)	928 (43.79)	1016 (47.95)	1024 (48.32)
**2012/13**	693 (43.92)	645 (40.87)	662 (41.95)	653 (41.38)	694 (43.98)	785 (49.75)
**Total**	6996 (46.24)	6755 (44.65)	6543 (43.24)	6559 (43.33)	7003 (46.28)	7574 (50.06)

AHA Aggressive: AHA guidelines (assuming that guideline recommends OAC at CHA_2DS_2-VASc = 1), AHA Conservative: AHA guidelines (assuming that guideline recommends non-OAC at CHA_2DS_2-VASc = 1), Casciano: Casciano tool, CHEST: CHEST guidelines, ESC: ESC guidelines, LaHaye: LaHaye tool.

Table S6. Baseline characteristics of patients who are discordant with OAC recommendations of decision aids.

Characteristics	OAC unexposed (n = 7461)					
	**AHA Aggressive recommends OAC, n (%)**	**AHA Conservative recommends OAC, n (%)**	**Casciano recommends OAC, n (%)**	**CHEST recommends OAC, n (%)**	**ESC recommends OAC, n (%)**	**LaHaye recommends OAC, n (%)**
**Total**	**6192 (82.99)**	**4473 (59.95)**	**4319 (57.8)**	**5035 (67.48)**	**6145(82.63)**	**1081 (14.49)**
**Female**	3089 * (49.89)	2450 * (54.77)	1895 * (43.88)	2252 * (44.73)	3089 * (50.11)	783 * (72.43)
**Age (18–30)**	53 * (0.86)	8 * (0.18)	21 * (0.49)	23 * (0.46)	53 * (0.86)	4 * (0.37)
**Age (31–49)**	666 * (10.76)	224 * (5.01)	383 * (8.87)	429 * (8.52)	660 * (10.71)	27 * (2.5)
**Age (50–64)**	2406 * (38.86)	1306 * (29.2)	1549 * (35.86)	1823 * (36.21)	2385 * (38.69)	141 * (13.04)
**Age (65–74)**	1240 * (20.03)	1108 * (24.77)	802 * (18.57)	933 * (18.53)	1240 * (20.11)	123 * (11.38)
**Age (75–84)**	1429 * (23.08)	1429 * (31.95)	1218 * (28.2)	1429 * (28.38)	1429 * (23.18)	596 * (55.13)
**Age (85 or greater)**	398 * (6.43)	398 * (8.9)	346 * (8.01)	398 * (7.9)	398 * (6.46)	190 * (17.58)
**CHF**	884 * (14.28)	850 * (19)	775 * (17.94)	884 * (17.56)	884 * (14.34)	477 * (44.13)
**Diabetes**	1265 * (20.43)	1222 * (27.32)	1088 * (25.19)	1265 * (25.12)	1265 * (20.52)	486 * (44.96)
**Vascular**	2202 * (35.56)	2063 * (46.12)	1601 * (37.07)	1921 * (38.15)	2202 * (35.72)	815 * (75.39)
**Anemia**	834 * (13.47)	735 * (16.43)	263 * (6.09)	759 * (15.07)	830 * (13.46)	253 * (23.4)
**Renal failure**	432 * (6.98)	404 * (9.03)	144 * (3.33)	421 * (8.36)	428 * (6.94)	101 * (9.34)
**Hypertension**	4263 * (68.85)	3531 * (78.94)	3645 * (84.39)	4263 * (84.67)	4236 * (68.71)	842 *(77.89)
**Liver failure**	232 † (3.75)	182 * (4.07)	157 (3.64)	206 * (4.09)	221 (3.58)	25 (2.31)
**Stroke**	476 * (7.69)	476 * (10.64)	476 * (11.02)	476 * (9.45)	476 * (7.72)	249 * (23.03)
**Bleeding**	664 * (10.72)	554 * (12.39)	373 * (8.64)	591 * (11.74)	646 * (10.48)	75 * (6.94)
**Alcohol**	87 † (1.41)	44 * (0.98)	59 (1.37)	72 (1.43)	82 * (1.33)	1 * (0.09)
**Anti-platelets**	401 * (6.48)	393 * (8.79)	330 * (7.64)	387 * (7.69)	401 * (6.5)	76 † (7.03)
**NASIDs**	1048 * (16.93)	772 † (17.26)	714 (16.53)	861 † (17.1)	1031 † (16.72)	79 * (7.31)
**GI protectants**	1271 * (20.53)	1019 * (22.78)	863 † (19.98)	1100 * (21.85)	1261 * (20.45)	254 * (23.5)
**Heparin**	23 † (0.37)	18 (0.4)	9 (0.21)	20 (0.4)	23 (0.37)	3 (0.28)
**Immunosuppressant**	25 (0.4)	16 (0.36)	9 (0.21)	20 (0.4)	24 (0.39)	5 (0.46)
**Corticosteroids**	820 * (13.24)	612 † (13.68)	543 (12.57)	667 † (13.25)	817 * (13.25)	155 (14.34)
**CHA_2DS_2-VASc = 0**	0 (0)	0 (0)	0 (0)	0 (0)	0 (0)	0 (0)

Table S6. *Cont.*

**Characteristics**	**OAC unexposed (n = 7461)**					
	**AHA Aggressive recommends OAC, n (%)**	**AHA Conservative recommends OAC, n (%)**	**Casciano recommends OAC, n (%)**	**CHEST recommends OAC, n (%)**	**ESC recommends OAC, n (%)**	**LaHaye recommends OAC, n (%)**
**CHA_2DS_2-VASc = 1**	1719 * (27.76)	0 (0)	721 * (16.69)	809 * (16.07)	1692 * (27.45)	0 (0)
**CHA_2DS_2-VASc = 2**	1420 * (22.93)	1420 * (31.75)	1017 * (23.55)	1194 * (23.71)	1420 * (23.03)	84 * (7.77)
**CHA_2DS_2-VASc = 3**	1089 * (17.59)	1089 * (24.35)	894 * (20.7)	1068 * (21.21)	1089 * (17.66)	6 * (0.56)
**CHA_2DS_2-VASc = 4**	868 * (14.02)	868 * (19.41)	714 * (16.53)	868 * (17.24)	868 * (14.08)	116 (10.73)
**CHA_2DS_2-VASc = 5**	596 * (9.63)	596 * (13.32)	512 * (11.85)	596 * (11.84)	596 * (9.67)	512 * (47.36)
**CHA_2DS_2-VASc = 6**	298 * (4.81)	298 * (6.66)	263 * (6.09)	298 * (5.92)	298 * (4.83)	205 * (18.96)
**CHA_2DS_2-VASc = 7**	141 * (2.28)	141 * (3.15)	137 * (3.17)	141 * (2.8)	141 * (2.29)	114 * (10.55)
**CHA_2DS_2-VASc = 8**	53 * (0.86)	53 * (1.18)	53 * (1.23)	53 * (1.05)	53 * (0.86)	38 * (3.52)
**CHA_2DS_2-VASc = 9**	8 (0.13)	8 † (0.18)	8 † (0.19)	8 † (0.16)	8 (0.13)	6 * (0.56)
**HAS-BLED = 0**	730 * (11.79)	90 * (2.01)	99 (2.29)	102 * (2.03)	730 * (11.84)	90 * (8.33)
**HAS-BLED = 1**	2114 * (34.14)	1306 * (29.2)	1561 * (36.14)	1681 * (33.39)	2114 * (34.29)	138 * (12.77)
**HAS-BLED = 2**	2041 * (32.96)	1797 * (40.17)	1678 * (38.85)	1951 * (38.75)	2041 * (33.11)	404 * (37.37)
**HAS-BLED = 3**	924 * (14.92)	898 * (20.08)	707 * (16.37)	918 * (18.23)	898 * (14.57)	392 * (36.26)
**HAS-BLED = 4**	288 * (4.65)	287 * (6.42)	202 * (4.68)	288 * (5.72)	287 * (4.66)	57 † (5.27)
**HAS-BLED = 5**	83 * (1.34)	83 * (1.86)	63 * (1.46)	83 * (1.65)	83 * (1.35)	0 (0)
**HAS-BLED = 6**	10 (0.16)	10 † (0.22)	7 (0.16)	10 (0.2)	10 (0.16)	0 (0)
**HAS-BLED = 7**	2 (0.03)	2 (0.04)	2 (0.05)	2 (0.04)	2 (0.03)	0 (0)

* Indicates statistically significant difference at $p < 0.001$. † Indicates statistically significant difference at $p < 0.05$. AHA Aggressive: AHA guidelines (assuming that guideline recommends OAC at CHA_2DS_2-VASc = 1), AHA Conservative: AHA guidelines (assuming that guideline recommends non-OAC at CHA_2DS_2-VASc = 1), Casciano: Casciano tool, CHEST: CHEST guidelines, ESC: ESC guidelines, LaHaye: LaHaye tool.

Table S7. Baseline characteristics of patients who are discordant with non-OAC recommendations of the decision aids.

**Characteristics**	**OAC exposed (n = 7668)**					
	**AHA Aggressive recommends non-OAC, n (%)**	**AHA Conservative recommends non-OAC, n (%)**	**Casciano recommends non-OAC, n (%)**	**CHEST recommends non-OAC, n (%)**	**ESC recommends non-OAC, n (%)**	**LaHaye recommends non-OAC, n (%)**
**Total**	**804 (10.49)**	**2282 (29.76)**	**2224 (29.00)**	**1524 (19.87)**	**838 (10.93)**	**6493 (84.68)**
**Female**	0 (0)	249 * (10.91)	692 * (31.12)	390 * (25.59)	1 * (0.12)	1897 * (29.22)
**Age (18–30)**	20 * (2.49)	26 * (1.14)	21 * (0.94)	21 * (1.38)	20 * (2.39)	29 † (0.45)
**Age (31–49)**	188 * (23.38)	409 * (17.92)	296 * (13.31)	250 * (16.4)	194 * (23.15)	558 * (8.59)
**Age (50–64)**	596 * (74.13)	1668 * (73.09)	1217 * (54.72)	910 * (59.71)	622 * (74.22)	3215 * (49.51)
**Age (65–74)**	0 (0)	179 * (7.84)	499 (22.44)	343 (22.51)	2 * (0.24)	1578 * (24.3)
**Age (75–84)**	0 (0)	0 (0)	175 * (7.87)	0 (0)	0 (0)	959 * (14.77)
**Age (85 or greater)**	0 (0)	0 (0)	16 * (0.72)	0 (0)	0 (0)	154 * (2.37)
**CHF**	0 (0)	128 * (5.61)	156 * (7.01)	0 (0)	0 (0)	898 * (13.83)
**Diabetes**	0 (0)	60 * (2.63)	214 * (9.62)	0 (0)	0 (0)	1285 * (19.79)
**Vascular**	0 (0)	101 * (4.43)	489 * (21.99)	203 * (13.32)	1 * (0.12)	1555 * (23.95)
**Anemia**	18 * (2.24)	76 * (3.33)	511 * (22.98)	45 * (2.95)	21 * (2.51)	537 * (8.27)
**Renal failure**	7 * (0.87)	35 * (1.53)	311 * (13.98)	12 * (0.79)	17 * (2.03)	408 (6.28)
**Hypertension**	0 (0)	761 * (33.35)	615 * (27.65)	0 (0)	30 * (3.58)	4251 * (65.47)
**Liver failure**	12 † (1.49)	56 (2.45)	70 (3.15)	32 † (2.1)	19 (2.27)	238 * (3.67)
**Stroke**	0 (0)	0 (0)	0 (0)	0 (0)	0 (0)	287 * (4.42)
**Bleeding**	44 * (5.47)	143 * (6.27)	257 * (11.56)	88 * (5.77)	64 (7.64)	612 * (9.43)
**Alcohol**	20 † (2.49)	62 * (2.72)	42 (1.89)	27 (1.77)	28 * (3.34)	123 * (1.89)
**Anti-platelets**	4 * (0.5)	17 * (0.74)	82 * (3.69)	20 * (1.31)	5 * (0.6)	390 † (6.01)
**NASIDs**	108 * (13.43)	367 * (16.08)	390 (17.54)	237 * (15.55)	135 † (16.11)	1302 * (20.05)
**GI protectants**	78 * (9.7)	289 * (12.66)	400 † (17.99)	191 * (12.53)	87 * (10.38)	1222 * (18.82)
**Heparin**	6 (0.75)	16 (0.7)	23 (1.03)	14 (0.92)	6 (0.72)	47 † (0.72)
**Immunosuppressant**	2 (0.25)	4 (0.18)	12 (0.54)	2 (0.13)	2 (0.24)	22 (0.34)
**Corticosteroids**	93 † (11.57)	273 * (11.96)	336 (15.11)	203 (13.32)	99† (11.81)	944 (14.54)
**CHA_2DS_2-VASc = 0**	804 (100)	804 (35.23)	804 (36.15)	804 (52.76)	804 (95.94%)	804 (12.38)

Table S7. *Cont.*

**Characteristics**	**OAC exposed (n = 7668)**					
	**AHA Aggressive recommends non-OAC, n (%)**	**AHA Conservative recommends non-OAC, n (%)**	**Casciano recommends non-OAC, n (%)**	**CHEST recommends non-OAC, n (%)**	**ESC recommends non-OAC, n (%)**	**LaHaye recommends non-OAC, n (%)**
**CHA_2DS_2-VASc = 1**	0 (0)	1478 * (64.77)	616 * (27.7)	529 * (34.71)	34 * (4.06)	1478 * (22.76)
**CHA_2DS_2-VASc = 2**	0 (0)	0 (0)	326 * (14.66)	166 * (10.89)	0 (0)	1493 * (22.99)
**CHA_2DS_2-VASc = 3**	0 (0)	0 (0)	235 * (10.57)	25 * (1.64)	0 (0)	1471 * (22.66)
**CHA_2DS_2-VASc =4**	0 (0)	0 (0)	161 * (7.24)	0 (0)	0 (0)	1024 † (15.77)
**CHA_2DS_2-VASc = 5**	0 (0)	0 (0)	62 * (2.79)	0 (0)	0 (0)	89 * (1.37)
**CHA_2DS_2-VASc = 6**	0 (0)	0 (0)	20 * (0.9)	0 (0)	0 (0)	109 * (1.68)
**CHA_2DS_2-VASc = 7**	0 (0)	0 (0)	0 (0)	0 (0)	0 (0)	12 * (0.18)
**CHA_2DS_2-VASc = 8**	0 (0)	0 (0)	0 (0)	0 (0)	0 (0)	9 * (0.14)
**CHA_2DS_2-VASc = 9**	0 (0)	0 (0)	0 (0)	0 (0)	0 (0)	4 † (0.06)
**HAS-BLED = 0**	627 (77.99)	1030 (45.14)	914 (41.10)	907 (59.51)	627 (74.32)	1030 (15.86)
**HAS-BLED = 1**	160 * (19.9)	926 * (40.58)	602 * (27.07)	495 (32.48)	160 * (19.09)	2237 * (34.45)
**HAS-BLED = 2**	16 * (1.99)	291 * (12.75)	391 * (17.58)	108 * (7.09)	16 * (1.91)	2147 (33.07)
**HAS-BLED = 3**	1 * (0.12)	32 * (1.4)	221 * (9.94)	13 * (0.85)	32 * (3.82)	702 * (10.81)
**HAS-BLED = 4**	0 (0)	3 * (0.13)	84 (3.78)	1 * (0.07)	3 * (0.36)	300 (4.62)
**HAS-BLED =5**	0 (0)	0 (0)	12 (0.54)	0 (0)	0 (0)	66 * (1.02)
**HAS-BLED = 6**	0 (0)	0 (0)	0 (0)	0 (0)	0 (0)	11 (0.17)
**HAS-BLED= 7**	0 (0)	0 (0)	0 (0)	0 (0)	0 (0)	0 (0)

* Indicates statistically significant difference at $p < 0.001$. † Indicates statistically significant difference at $p < 0.05$. AHA Aggressive: AHA guidelines (assuming that guideline recommends OAC at CHA_2DS_2-VASc = 1), AHA Conservative: AHA guidelines (assuming that guideline recommends non-OAC at CHA_2DS_2-VASc = 1), Casciano: Casciano tool, CHEST: CHEST guidelines, ESC: ESC guidelines, LaHaye: LaHaye tool.
